# Combined Effect of Citric Acid and Polyphenol-Rich Grape Seed Extract towards Bioactive Smart Food Packaging Systems

**DOI:** 10.3390/polym15143118

**Published:** 2023-07-21

**Authors:** Akvilė Pažarauskaitė, Estefanía Noriega Fernández, Izumi Sone, Morten Sivertsvik, Nusrat Sharmin

**Affiliations:** 1 Department of Chemistry, Bioscience and Environmental Engineering, University of Stavanger, Richard Johnsensgate 4, 4021 Stavanger, Norway; akvile.pazarauskaite@gmail.com; 2 Department of Processing Technology, Nofima AS, Richard Johnsensgate 4, 4021 Stavanger, Norway; estefania.noriegafernandez@efsa.europa.eu (E.N.F.); izumi.sone@nofima.no (I.S.); morten.sivertsvik@nofima.no (M.S.); 3 European Food Safety Authority, Via Carlo Magno 1A, 43126 Parma, Italy; 4 Department of Food Safety and Quality, Nofima AS, Osloveien 1, 1430 Ås, Norway

**Keywords:** sodium alginate, citric acid, grape seed extract, active packaging

## Abstract

Alginate films (2% w·v^−1^) were prepared with varying concentrations (5–20% *w*/*w*) of citric acid and aqueous grape seed extract (GSE) filtrate (11.66 ± 1.32 g GAE/L) using the solvent-evaporation method. Crosslinking alginate via ester bonds (FTIR analysis) with citric acid up to 10% (*w*/*w*) led to a 33% increase in tensile strength, a 34% reduction in water vapor transmission rate (WVTR), and had no impact on elongation at break. Crosslinking alginate with citric acid in the presence of GSE increased the tensile strength by 17%, decreased WVTR by 21%, and significantly improved DPPH scavenging activity. Moreover, after incubation for 24 h at 37 °C, the film-forming solutions exhibited increased antimicrobial activity, resulting in 0.5- and 2.5-log reductions for *Escherichia coli* and *Staphylococcus aureus*, respectively, compared to the values obtained without the addition of GSE. The stronger inhibitory effect observed against Gram-positive bacteria can be attributed to the unique composition and structure of their cell walls, which creates a barrier that restricts the penetration of polyphenols into the cells. The pH adjustment of the GSE film-forming solution from 2.0 to 10.0 shifted the UV/VIS absorption spectra, resulting in a colour change from yellow to red. The findings of this study have showcased the potential of combining GSE and citric acid to enhance the functionality and bioactivity of alginate films for applications in smart food packaging.

## 1. Introduction

The growing recognition of the environmental impact of traditional plastics and the fast-paced advancements in food innovation have shifted the focus towards smart (active and intelligent systems) biopolymer-based food packaging materials, in a world that seeks more sustainable and diversified food systems.

In this study, the base biopolymer used for developing smart food packaging material was sodium alginate, which is naturally found in seaweeds, is readily available, and has high stability, distinct colloidal and good gelling properties, great biocompatibility and non-toxicity, as well as the ability to be chemically or biochemically altered [1,2,3,4]. However, pure alginate films are water soluble and have poor barrier, antioxidant, and antimicrobial properties [4,5,6,7]. Improvements in the three-dimensional biopolymer structures can be introduced by crosslinking, which stabilizes the biopolymer mesh, reduces the water vapor/gas permeability, and improves mechanical strength [8,9,10]. For instance, citric acid is known to facilitate the crosslinking of hydroxyl groups within sodium alginate polymers, and as a result the covalent intermolecular di-ester links are created between hydroxyl groups of the polysaccharide and the carboxyl groups of the crosslinking agents [11]. In addition, citric acid acts as an antioxidant synergist [12,13] and has been shown to inhibit the growth of foodborne pathogens [14,15,16,17].

Active and visually responsive food packaging materials able to monitor/flag pH changes during food storage would confer a competitive advantage towards food safety [18,19]. However, the synthesis and degradation of traditional synthetic pH dyes (e.g., methyl and cresol red, chlorophenol, bromocresol green and purple, etc.) may lead to the formation of harmful substances and therefore public health and environmental concerns [20,21,22]. Hence, natural bioactives and pigments are being researched as alternative sources for smart food packaging applications, with premium nutritional/sensory quality and prolonged shelf-life. Phenolic compounds, known for their diverse biological activity and characteristic colour changes in response to pH variations, show great potential for applications in smart food packaging and edible coatings [23]. In particular, the antioxidant activity of polyphenolic-rich grape (*Vitis vinifera*) seed extracts (GSE) has been attributed to flavonoids, which can scavenge free radicals, have metal-chelating capabilities, and reduce the formation of hydroperoxide [24,25,26]. Moreover, the antimicrobial activity of GSE films has been reported over a broad spectrum of pathogenic bacteria [27,28,29,30]. Interestingly, colour response as a result of changing pH reported in GSE has been attributed to the phenolic content [31,32].

The main aim of this study was to develop a visually responsive alginate-based active food packaging system with good antioxidant and antimicrobial properties without affecting the inherent functional properties of the alginate film. To do so, GSE as an active and intelligent agent and citric acid as a crosslinking agent were added to the film-forming solution. Initially, different citric acid amounts were added to the alginate solution to determine the best alginate–citric acid combination to achieve the best functional properties in terms of mechanical and barrier properties. Finally, GSE was incorporated into alginate–citric acid films and the antioxidant, antimicrobial, and colour-changing properties were evaluated.

## 2. Materials and Methods

### 2.1. Materials

Sodium alginate (alginic acid sodium salt from brown algae, low viscosity), citric acid (99%, 192.12 g/mol), sodium hypophosphite monohydrate (≥99%, 105.99 g/mol), Folin–Ciocalteu’s phenol reagent (1.240 g/cm^3^), and gallic acid (97.5–102.5% titration, 170.12 g/mol) were purchased from Sigma-Aldrich (Darmstadt, Germany). Vegetable capsules of grape (*Vitis vinifera*) seed extract (GSE) were acquired from Swanson Health Products (Fargo, ND, USA). Sodium hydroxide solution (1 and 0.1 mol/L), hydrochloric acid (1 and 0.1 mol/L), sodium hydrogen carbonate (analysis grade, 84.01 g/mol), Plate Count Agar (PCA) and sodium chloride (analysis grade, 58.44 g/mol) were purchased from Merck (Darmstadt, Germany). DPPH powder (2,2-Diphenyl-1-picrohydrazyl free radical, 95%) was acquired from Alfa Aesar (Kandel, Germany). The 96% ethanol (rectified alcohol) was purchased from Antibac (Asker, Norway). Mueller-Hinton Agar (MHA) and Tryptone Soy Broth (TSB) were acquired from Oxoid (Basingstoke, UK).

### 2.2. Preparation of GSE Filtrates

Aqueous GSE filtrates were prepared as described by Silván et al. (2013) [33], with slight modifications. Vegetable capsules of GSE powder (0.377 g powder each) were stored at room temperature under 95% vacuum (FH-S SuperMAX C V3, Webomatic, Bochum, Germany) and thereafter handled under aseptic conditions. A total of 2 g of GSE powder was transferred to a 50 mL Falcon tube (Sarstedt, Numbrecht, Germany) with 40 mL sterile distilled water (DW). After 5 min vortexing at room temperature, the suspension was centrifuged at 7000 rpm and 4 °C for 10 min (Heraeus Multifuge X3 FR centrifuge, Thermo Fisher Scientific, Braunschweig, Germany). The supernatant was transferred to a 50 mL Falcon tube and centrifuged under the above-mentioned conditions. The resulting supernatant was filter-sterilised through a 0.45 μm pore size membrane filter (Labolytic, Trondheim, Norway) using a Buchner filtration system coupled to a vacuum/pressure piston pump (VWR VCP80, VWR International, Leuven, Belgium). The GSE filtrates were stored at 4 °C for up to 6 h before use.

### 2.3. Preparation of Film-Forming Solutions

The film-forming solutions were prepared following a modified version of the method described by Sharmin et al. (2021) [11].

To make an alginate film-forming solution (alg), 2.0 g sodium alginate was dissolved in 100 mL distilled water (DW) by stirring for 1 h at 500 rpm and room temperature using a magnetic stirrer (MR Hei-Tec magnetic stirrer, Heidolph Instruments, Schwabach, Germany).

For alginate and citric acid films, at first, alginate was homogenised with DW by stirring for 1 h at 500 rpm. Afterwards, sodium hypophosphite monohydrate (2.5, 5.0, 7.5, and 10.0% *w*/*w* alginate) and citric acid (5, 10, 15, and 20% *w*/*w* alginate) at the respective concentrations were stirred for 2 h at 500 rpm and room temperature (Table 1).

At 4 °C, aqueous GSE filtrates were diluted 1:2 in sterile DW. Then, 100 mL of the diluted solution was used to dissolve 2.0 g alginate by stirring for 1 h at 500 rpm (GSE_alg). Afterwards, 1.0 g sodium hypophosphite monohydrate and 0.2 g citric acid were added (GSE_alg_ca10). The resulting solution was stirred for 2 h at 500 rpm and 4 °C.

The composition of film-forming solutions is presented in Table 1. Film-forming solutions were used as samples for FTIR analysis and antimicrobial assays.

### 2.4. Preparation of Films

Films were prepared with the casting/solvent-evaporation method. To prepare the films, 20 mL of film-forming solution was poured out into polystyrene Petri dishes with a diameter of 90 mm (Sigma-Aldrich, Darmstadt, Germany) and set aside to dry. The films were dried without the Petri dish lid either at room temperature or at 12 °C until the liquid evaporated. The film was retrieved from the dish when the whole surface area became firm enough (approximately 3 days at room temperature and 5 days at 12 °C). The samples stored at 12 °C were additionally left at room temperature on a bench in a Petri dish with the lid on for overnight water evaporation. While the room temperature drying occasionally led to the adherence of the film to the dish surface, all films prepared at 12 °C were retrieved satisfactorily. The mechanical, barrier, and antioxidant properties of the films were evaluated and no significant differences were found for the films dried using one method or the other.

### 2.5. Mechanical Properties

To determine the mechanical properties (tensile strength, elongation at break, and tensile modulus), the films were cut into 60 mm × 15 mm (length × width) rectangular pieces [11] right after the whole surface area of a film became firm enough to remove it from the plate. The strips of films dried at 12 °C were weighted from the top to avoid coiling (in other words, to ensure straightness), and left overnight on the bench to stabilize the structure. The thickness was measured with a 0.01 mm resolution digital calliper. TA.XT Plus texture analyser with Exponent software (Stable Micro Systems Ltd., Godalming, UK) was used to measure the maximum force required to break the film and the change in length at the point of rupture. A 50 kg load cell was attached to the texture analyser, the span distance between tensile grips was fixed at 25 mm, and the test speed was set at 1 mm/s [34]. More than six pieces of each film type were tested.

### 2.6. FTIR

The FTIR spectra of the samples were measured using the Bruker INVENIO^®^ Spectrometer (Bruker Optics, Leipzig, Germany), which was equipped with a high-throughput extension HTS-XT for precise analysis. The OPUS v8.5 software was employed to control the data acquisition process. Spectra were acquired in transmission mode, covering a range of 4000–600 cm^−1^ (4 cm^−1^ resolution). To establish a baseline, a background spectrum of the empty 96-well Silicon plate was collected before each sample measurement. The background scans were repeated 40 times. Subsequently, the 5 replicate spectra of samples were averaged and baseline corrected using the Unscrambler^TM^ v11 software from Camo Analytics AS (Oslo, Norway).

### 2.7. Barrier Properties

Water vapor transmission rate (WVTR) was used to evaluate the water molecules’ permeability through the barrier of the film. The method described by Sarwar et al. (2018) [35] was slightly adjusted to work with sodium alginate films. A glass test tube with an inner diameter (*d*) of 13.5 mm was filled with 10 mL DW and the opening was closed with 15 mm × 15 mm film fragments tightened using Laboratory Parafilm PM996 sealing film (American National Can, Chicago, IL, USA). Test tubes with samples were weighted using Fisher Scientific Precision Series balance (Thermo Fisher Scientific, Waltham, MA, USA) before (*W_i_*) and after (*W_t_*) heating in a 45 °C oven for 24 h (*t*). More than four replicates of each film composition were tested. The WVTR was determined by Equation (1):

(1) $$WVTR=\frac{W_{i}-W_{t}}{A}\cdot t$$

WVRT is the water vapor transmission rate (g/m^2^h), *W_i_* is the initial weight of the test tube (g), *W_t_* is the final weight of the test tube (g), *A* is the area of tube opening (0.25 ∙ π ∙ *d*^2^) (m^2^), and *t* is 24 h.

### 2.8. Total Phenolic Content

The Folin–Ciocalteu micro-method [33,36] was used, with slight modifications, to determine the total phenolic content (TPC) of aqueous GSE filtrates prepared at 50 mg GSE/mL (2 g GSE powder in 40 mL DW).

Then, 10 μL sample aliquots at an adequate dilution were added in triplicate to a 96-well microplate (Greiner Bio-One, Frickenhausen, Germany) followed by 150 μL of the Folin–Ciocalteu solution (0.5 mL reagent in 7 mL DW). After 3 min incubation, each well of the plate was filled with 50 μL of sodium bicarbonate solution (0.96 g in 10 mL DW, filtered through 11 μm pore size Whatman filter paper and diluted 2:3 in DW) and subsequently the plate was carefully kept in a dark environment at room temperature for 2 h. Absorbance was measured at 725 nm using a Synergy H1 multi-mode reader and Gen5 software for data acquisition (BioTek Instruments, Winooski, VT, USA). Gallic acid was the standard used for the calibration curve.

### 2.9. Antioxidant Properties

DPPH radical scavenging activity was measured as described by Dysjaland et al. (2022) [37], with some modifications. The film was dissolved in DW to a concentration range of 0.01–3.00 mg/mL sodium alginate. A 0.15 mM DPPH and 96% ethanol solution was stirred for 15 min in a darkened flask and stored at 4 °C for at least 1 h before use. GSE_alg and GSE_alg_ca10 solutions were centrifuged at 7000 rpm and 4 °C for 10 min to obtain a clear supernatant. The sample was mixed with an equal part of the DPPH solution, and kept in the dark at room temperature for 1 h. The absorbance of the mixture was measured at 517 nm in triplicate using a multi-mode spectrophotometer (Synergy H1, BioTek Instruments, Winooski, VT, USA). DPPH solution was used as a control, and the blank was DW. DPPH radical scavenging activity (%) was expressed using Equation (2):

(2) $$DPPH\;scavenging\;activity=\left(1-\frac{{Abs}_{S}-{Abs}_{B}}{{Abs}_{C}-{Abs}_{B}}\right)\cdot 100\%$$

*Abs_S_* is the absorbance of the sample, *Abs_B_* is the absorbance of the blank (DW), and *Abs_C_* is the absorbance of the control (0.15 mmol/L DPPH solution).

### 2.10. Antimicrobial Properties

The evaluation of antimicrobial properties of the film-forming solutions was conducted according to the modified method described by Dysjaland et al. (2022) [37] and Sharmin et al. (2021) [38]. The antimicrobial studies focused on *Escherichia coli* CCUG 10979 (Gram-negative) and *Staphylococcus aureus* CCUG 1828 (Gram-positive) reference strains, obtained from the Culture Collection of the University of Gothenburg (Sweden). These strains were stored in Microbank^TM^ porous beads (Microbank, Pro-lab Diagnostics, Richmond Hill, ON, Canada) at −80 °C.

To initiate the antimicrobial studies, *E. coli* and *S. aureus* microbank beads were streaked onto PCA plates and incubated overnight at 37 °C in a Sanyo MIR-154 incubator (SANYO Commercial Solutions, Kennesaw, GA, USA). Subsequently, a singled-out colony was transferred into 5 mL of Tryptic Soy Broth (TSB) and incubated at 37 °C for 24 h. The stationary-phase cultures were diluted in TSB to inoculate the film-forming solutions (≈10^7^ CFU/mL).

For the antimicrobial studies, 250 µL of the selected film-forming solutions or 250 µL of TSB as control were added to 1.5 mL Eppendorf tubes along with 50 µL of the corresponding TSB-diluted *E. coli* or *S. aureus* cell suspensions. The Eppendorf tubes were incubated at 37 °C and 300 rpm for 24 h using a VorTemp 56 incubator (Labnet International, Edison, NJ, USA) to simulate temperature abuse conditions. Viable plate counts were determined for both the test and control samples immediately after inoculation and following the incubation period. The pH measurements were performed using a pH electrode LE422 and FiveEasy Plus pH-meter (Mettler-Toledo, Columbus, OH, USA).

### 2.11. Colour Characteristics

The effect of pH on the colour of GSE_alg_ca10 film-forming solutions containing diluted GSE filtrates (1:2 in DW) was assessed. The pH of film-forming solutions was measured using glass electrode LE410 with a temperature sensor for aqueous samples (pH-meter FiveEasy Plus, Mettler-Toledo, Columbus, OH, USA). The pH was adjusted to 2.0, 4.0, 6.0, 8.0, or 10.0 with either sodium hydroxide (0.1 or 1.0 M) or hydrochloric acid (0.1 or 1.0 M), and the solutions were then centrifuged at 7000 rpm and 4 °C for 10 min. Then, 250 μL of clear supernatant was pipetted in triplicate in a 96-well microplate (Greiner Bio-One, Frickenhausen, Germany). UV/VIS absorption spectra for the above-mentioned GSE solutions were recorded at 230–800 nm wavelength with 10 nm steps using a Synergy H1 multi-mode reader and Gen5 data acquisition software (BioTek Instruments, Winooski, VT, USA).

### 2.12. Statistical Analyses

The experiments were performed in triplicate or more (specific number of replicates is mentioned for each experiment). Statistical analysis was carried out using the *T*-test and analysis of variance (ANOVA) in SPSS software (SPSS statistics, version 26, IBM, Chicago, IL, USA). Tukey’s multiple comparison tests were used as a post hoc test, and a significance level of *p* < 0.05 was considered statistically significant. It was assumed that the variances were equal. The graphical data are presented as the mean value along with the standard deviation.

## 3. Results

### 3.1. Citric Acid Concentration

#### 3.1.1. Effect of Citric Acid Concentration on Mechanical Properties

**Figure 1:**
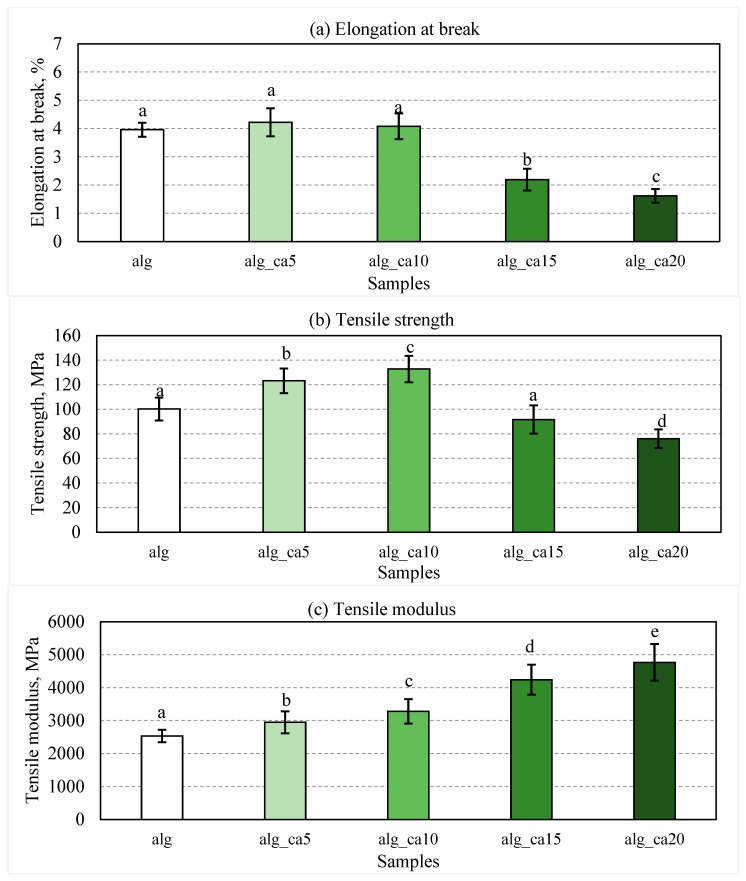
Mechanical properties of alginate–citric acid films: (**a**) elongation at break, (**b**) tensile strength, (**c**) tensile modulus. White columns depict controls (i.e., sodium alginate). Light to dark green columns display 5, 10, 15, and 20% citric acid addition to alginate films. Error bars represent standard deviation (*n* ≥ 5). Statistical significance (*p* < 0.05) is illustrated by different letters (a–e).

Alginate films were prepared with various citric acid concentrations (0, 5, 10, 15, and 20% *w*/*w* alginate), and their mechanical properties are presented in Figure 1.

No significant change in the elongation at break (Figure 1a) was observed for the alginate films containing 0, 5, and 10% citric acid. For further increases in the citric acid content, up to 20%, the elongation at break of the films dropped significantly (*p* < 0.001) as compared to the control alginate films. The clear decrease in the elongation at break could be caused by the crosslinking reactions, which connect the biopolymer molecules and, hence, reduce the mobility of the structure. Similar results were reported after crosslinking 1.5% (*w*/*w*) citric acid with starch/polyester polymers [39] or 1% (*w*/*w*) citric acid with peanut proteins [40]. The effect was attributed to the improved intermolecular connections, which limited the flexibility and increased rigidity.

Concerning the tensile strength as a function of the citric acid concentration (Figure 1b), the tensile strength of the films with 5% and 10% citric acid increased significantly by 23% (*p* < 0.001) and 33% (*p* < 0.001), respectively, as compared to the uncrosslinked control alginate films. With the further increase in the citric acid concentration from 10% to 15 and 20%, the tensile strength reduced significantly (*p* < 0.001) from 133 MPa to 92 and 76 MPa, respectively. It has been reported previously that citric acid can act as crosslinking agent by participating in intra- and inter-molecular interactions within the biopolymer reticulum, hence contributing to a more compact structure and thereby improved tensile strength up to certain citric acid concentrations [41,42,43,44]. However, the presence of extra/unreacted citric acid at higher concentrations may function as a plasticizing agent, which can reduce the interactions between the macromolecules, leading to tensile strength reduction [11,45,46]. In this study, the tensile strength of alginate–citric acid films increased with increasing citric acid content, and reached a maximum at 10% citric acid concentration, then declined with a further increase in the citric acid concentration.

The tensile modulus of the films with increasing citric acid concentrations increased throughout the experiment (Figure 1c). Such findings suggested that the citric acid functioned as a crosslinking agent, and thus film formulations with lower citric acid concentrations formed less rigid films due to a lower amount of crosslinked junctions. These results were in line with previous studies, which showed that a higher content of crosslinking points resulted in a high tensile modulus [47,48,49,50].

#### 3.1.2. FTIR and the Effect of Citric Acid

**Figure 2:**
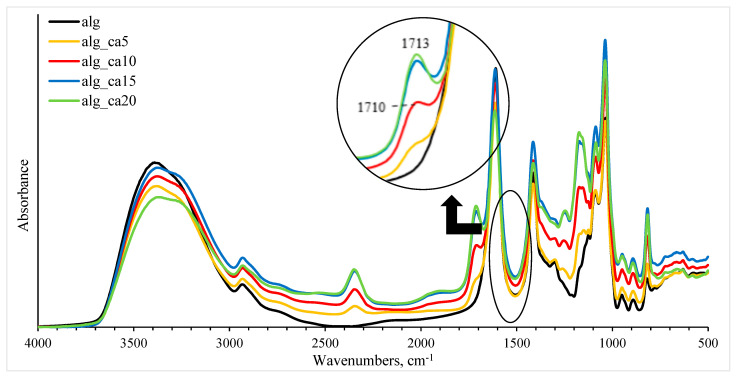
Intensity differences in FTIR spectra of alginate−citric acid samples presented by black (alg), yellow (alg_ca5), red (alg_ca10), blue (alg_ca15), and green (alg_ca20) colours.

Figure 2 shows the FTIR spectra of sodium alginate crosslinked with 0, 5, 10, 15, and 20% (*w*/*w* alginate) citric acid. When citric acid is incorporated into the alginate matrix, carboxylic groups of citric acid react with –OH groups on pyranose rings of the sodium alginate backbone [11,51]. A sharp absorption in the 1690–1750 cm^−1^ region is characteristic of the C=O group in citric acid [52]. In this study, the peak was found at around 1710 cm^−1^ in the samples containing citric acid, which was not present in pure alginate samples.

With increasing citric acid concentration, the characteristic peaks in the 1710–1713 cm^−1^ region increased in intensity and shifted towards higher wavenumbers. The bands could be assigned to the overlapping peaks from ester bonds joining biopolymer chains with citric acid and unreacted carboxyl groups of citric acid [16,39,41,45,53]. The effect of the addition of organic acid to starch/poly-butylene-adipate-co-terephthalate (PBAT) films was studied and it was found that the peaks observed at 1715 cm^−1^ were an overlap of the ester bonds in the native PBAT molecules and the crosslinking points with the citric acid [39]. In addition, the peak at 1730 cm^−1^ was ascribed to ester bonds between wheat straw hemicellulose and citric acid, and the peak observed at 1717 cm^−1^ was ascribed to the free carboxylic acid groups [45]. Moreover, it has been determined that the intensity of the peaks observed at 1711–1722 cm^−1^ increased at higher citric acid concentrations in the thermoplastic starch matrix, and therefore the increment was related to the increasing degree of esterification [53]. The shift of the peaks towards higher vibrational wavelengths could also indicate the changes in microstructures [54]. For example, the crosslinked starch and citric acid films were washed of free citric acid and catalyst residues and the band at 1724 cm^−1^ was found in the FTIR spectra, confirming ester linkages between citric acid and starch [41]. However, it must be mentioned that the C=O stretch of the carboxylic acid on alginate at pH 1.2 has been determined at 1734 cm^−1^ for the alginate hydrogel blended with NaCl [55]. In this study, the lowest pH was 3.7 ± 0.1 (20% citric acid), and hence not all alginate molecules were protonated, but the acidification of the medium could have promoted the shift of wavenumbers towards the higher values.

Furthermore, the intensity of the peak at 3000–3600 cm^−1^ related to the –OH vibrations decreased with increasing citric content. Similar results were found for polyvinyl alcohol (PVA)/starch films crosslinked with 5–30% (*w*/*w*) citric content, and the results indicated that the –OH groups of biopolymers were consumed by citric acid to create ester linkages [16].

The peaks observed in the range of 1300–1000 cm^−1^ wavenumbers have been attributed to the C–O stretching vibrations [56]. Therefore, the shifting bands within the 1302–1126 cm^−1^ range may represent the changes in the alginate backbone and/or citric acid structure. Interestingly, with increasing citric acid concentration, the shift in the peaks towards lower wavenumbers of 822 cm^−1^ and increments in their amplitude were measured. This peak had been previously assigned to the mannuronic acids in the alginate structure [57]. Hence, these residues might have been affected by crosslinking. However, the 812 cm^−1^ band has also been observed in the FTIR spectra of SHP [58]. In this work, the SHP concentration increased with increasing citric acid concentration, which could have influenced the already-mentioned changes.

#### 3.1.3. Effect of Citric Acid on Barrier Properties

As presented in Table 1, the addition of 5, 10, 15, and 20% citric acid to alginate films caused a significant (*p* < 0.001) decrease in the WVTR compared to pure alginate films; in particular; WVTR was reduced by 29, 34, 36, and 44%, respectively. However, between 5 and 20% citric acid samples, only the WVTR of 20% citric acid films was significantly reduced (*p* ≤ 0.033). It had been reported that the WVTR of a film is dependent on the number of available polar groups of polymer chains [44]. Hence, the decrease in WVTR could be due to the replacement of hydrophilic –OH groups present in alginate with hydrophobic ester groups from citric acid [11,45]. Also, the addition of citric acid is likely to have created a complex path for water molecules to travel through, because a denser film structure with strong inter- and intra-molecular interactions was created by the crosslinking action [59].

### 3.2. GSE

#### 3.2.1. Effect of GSE on Mechanical Properties

**Figure 3:**
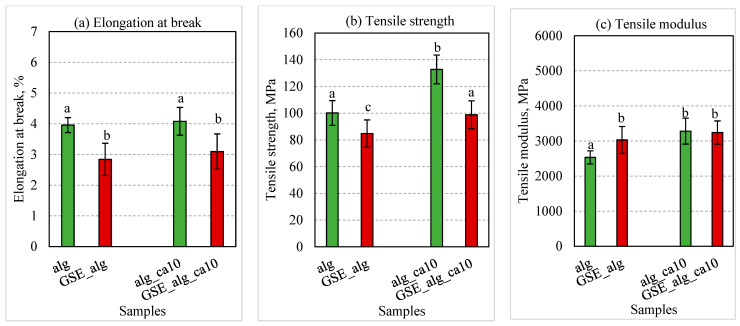
Mechanical properties of DW and GSE films: (**a**) elongation at break, (**b**) tensile strength, (**c**) tensile modulus. The green and red columns depict DW and GSE films, respectively. Column groups from left to right: without citric acid (alg, GSE_alg), with 10% citric acid (alg_ca10, GSE_alg_ca10). Error bars represent standard deviation (*n* ≥ 6). Statistical significance (*p* < 0.05) is illustrated by different letters (a−c).

The GSE was used to improve the antioxidant and antimicrobial properties of alginate films. Results of previously described experiments showed that a 10% citric acid addition to alginate films led to the highest tensile strength of homogenous films, and therefore this composition was chosen for GSE studies. The comparative results of elongation at break, tensile strength, and modulus of optimum film compositions prepared using DW and GSE solvents without citric acid (alg and GSE_alg, respectively) and with 10% citric acid (alg_ca10 and GSE_alg_ca10, respectively) are presented in Figure 3.

The results of the current study showed that GSE significantly affected the mechanical properties of alginate films. In particular, GSE_alg samples exhibited a significant decrease in tensile strength (*p* = 0.034) plus elongation at break (*p* < 0.001), while a significant increase in tensile modulus (*p* = 0.044) values was observed as compared to corresponding pure alginate films. The samples with polyphenolic GSE might be affected by the interactions of phenolic compounds with biopolymer molecules by hydrogen bonds and/or hydrophilic interactions, resulting in an increased tensile modulus and decreased elongation [60]. On the other hand, the decrease in the tensile strength could be the result of the weakened inter-molecular and physical polymer–polymer interactions leading to the breakage of the film [30]. Also, it is worth mentioning that the addition of the extract increased the solid content of the film, and GSE films were thicker than DW-alginate films (0.06 ± 0.01 and 0.04 ± 0.00 mm, correspondingly), indicating the expansion of the alginate matrix [56].

The tensile strength of GSE films was significantly improved (*p* = 0.015) by 14% with the addition of 10% citric acid (Figure 1b). The obtained results were in good agreement with a previous study by Ounkaew et al. (2018) [16], where polyvinyl alcohol/starch films enriched with an active compound (spent coffee ground) and citric acid content up to 10% increased the tensile strength compared to control samples without citric acid.

#### 3.2.2. FTIR and the Effect of GSE

**Figure 4:**
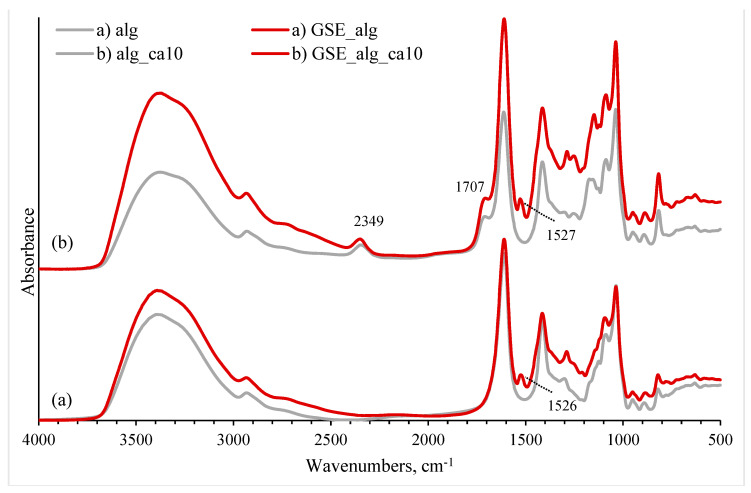
FTIR spectra of GSE−alginate and their combination with 10% citric acid. Bottom (a) spectra represent controls, i.e., alginate (alg) in grey and GSE−alginate (GSE_alg) in red. Top (b) spectra illustrate 10% citric acid addition to alginate (alg_ca10 in grey), and to GSE−alginate (GSE_alg_ca10 in red).

A new peak at 1526 cm^−1^ was observed in the FTIR spectra of alginate solutions containing GSE, which could be assigned to the vibration of the cyclobenzene ring of phenolic compounds (Figure 4) [61,62]. In the GSE_alg_ca10 sample, a peak at 1707 cm^−1^ peak was observed, indicating that crosslinking with citric acid happened in the GSE solution as well.

#### 3.2.3. Effect of GSE on Barrier Properties

In comparison with pure alginate films, the WVTR significantly decreased when GSE was incorporated into alginate-based films (*p* < 0.001; Table 1), which was in good agreement with previous studies [28,60,63]. Polyphenolic compounds present in GSE are characterized by the presence of one or more hydroxyl groups linked to aromatic rings [64]. The increased number of crosslinks via hydrogen and hydrophobic interactions in the polymeric matrix likely caused the decrease in WVTR in films containing GSE compared to native biopolymers [28]. The phenols in the GSE films interacted with some –OH groups of alginates and reduced the hydrophilic characteristics of the film matrix related to water vapor permeability [63].

The WVTR significantly decreased with the addition of 10% citric acid (*p* < 0.001). Such results indicate the crosslinking between alginate and citric acid. The WVTR of GSE_alg_ca10 was similar to alg_ca10 films (91 ± 5 and 92 ± 4 g/m^2^h, respectively).

#### 3.2.4. Effect of GSE on Antioxidant Properties

**Figure 5:**
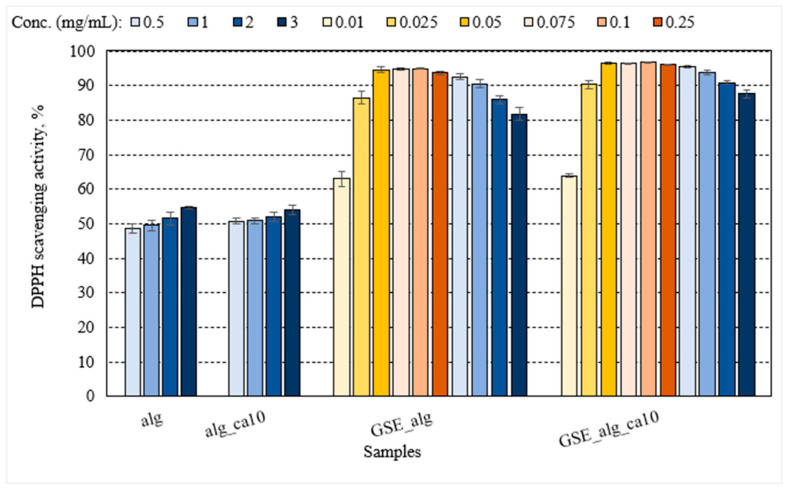
Free radical DPPH∙ scavenging activity of alginate films (alg) and their combination with GSE (GSE_alg); alginate films treated with 10% citric acid (alg_ca10) and their combination with GSE (GSE_alg_ca10). The darkening of the colour tone illustrates the increasing alginate concentration: 0.5, 1.0, 2.0, 3.0 mg/mL (from light to dark blue, respectively), 0.01, 0.025, 0.05 mg/mL (from light to dark yellow, respectively), and 0.075, 0.1, 0.25 mg/mL (from light to dark orange, respectively). The GSE_alg and GSE_alg_ca10 dilutions were recalculated to mg GAE/mL from highest to lowest: 0.557, 0.372, 0.186, 0.093, 0.046, 0.019, 0.014, 0.009, 0.005, 0.002 mg GAE/mL. Error bars represent standard deviation (*n* ≥ 3).

The addition of GSE into the alginate film significantly improved the DPPH scavenging activity, by 1.7-fold as compared to pristine alginate film regardless of the alginate concentration (0.5–3.0 mg/mL) (Figure 5). GSE is rich in polyphenols (TPC = 0.22 ± 0.03 g GAE/g), particularly in proanthocyanidins. Several biopolymer films have shown increased antioxidant properties upon GSE incorporation, including chitosan [60], fish skin gelatine [24], alginate [63], and pectin/pullulan blend films [26], as affected by GSE type, TPC, concentrations, and the method used in the analytical determination, as well as by potential contributions of the dominant biopolymer. Typically, DPPH scavenging activity in edible films increases with increasing concentrations of the antioxidant agent [30,63]. However, the DPPH scavenging activity of the GSE_alg and GSE_alg_ca10 samples decreased with the increasing GSE concentrations (0.093–0.557 mg GAE/mL). This could be attributed to the aggregation of GSE at high concentrations limiting the compatibility of the biopolymer and GSE, as observed in gelatin–GSE–glycerol films at high concentrations (5 mg/mL) [24]. Alternatively, lower DPPH activity detected in GSE_alg and GSE_alg_ca10 samples may be a result of reversibility in the DPPH reaction, where the DPPH∙ radical receives a hydrogen atom H and becomes colourless DPPH-H upon exposure to the antioxidant agent [65]. Due to the chemical equilibrium of the reaction solution, some of the reduced form DPPH-H could convert back into the free DPPH∙ radical. At low antioxidant concentrations, the re-conversion is small, and hence the effects on absorbance are negligible. However, at high concentrations of the antioxidant, the amount of re-converted DPPH∙ would be higher, which could lead to the underestimation of the antioxidant activity [65]. Considering this possibility, the DPPH scavenging capacity was measured at a wider range of low concentrations, 0.01–0.1 mg alginate/mL (0.002–0.019 mg GAE/mL) (Figure 5). The DPPH activity of GSE_alg and GSE_alg_ca10 films significantly increased with increasing concentrations between 0.01 and 0.05 mg alginate/mL (0.002–0.009 mg GAE/mL). However, there was no statistically significant increase in the DPPH activity with concentrations between 0.05 and 0.1 mg alginate/mL (0.009–0.019 mg GAE/mL). Regardless of GSE concentration, the presence of GSE in alginate films increased their antioxidant properties compared to pristine alginate films.

GSE–alginate antioxidant activity was significantly enhanced by crosslinking with 10% citric acid, although the effect size was small, ≈4%, *p* < 0.001 (Figure 5). Because of its ability to chelate metal ions [12], citric acid has been shown to increase the antioxidant properties of phenolic-rich extracts [13,66,67].

#### 3.2.5. Effect of GSE on Antimicrobial Properties

**Figure 6:**
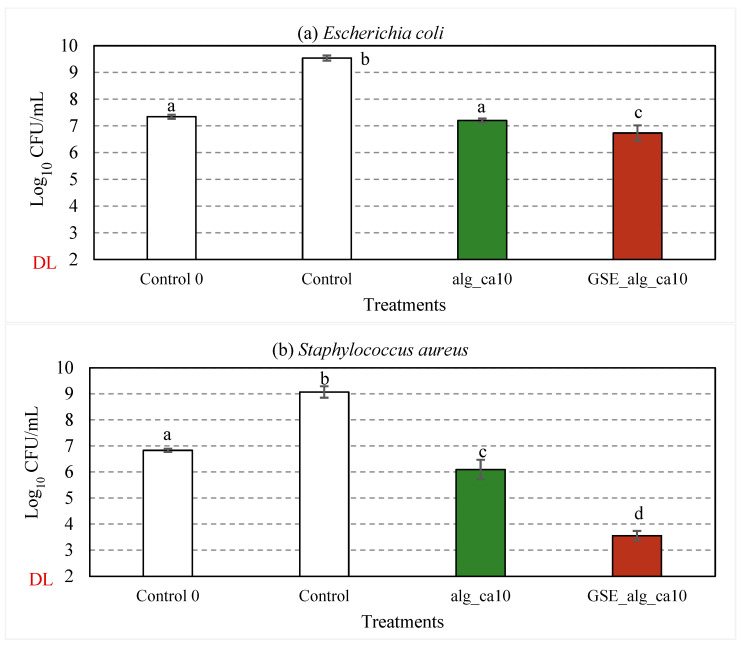
Effect of GSE (in red) on the inhibitory activity against *E. coli* (**a**) and *S. aureus* (**b**) of alginate and citric acid film-forming solutions (in green). In white, control samples (TSB). “Control 0” represents the average initial viable counts for all the assayed conditions (≈10^7^ CFU/mL). “DL” is the detection limit of the colony counting method (10^2^ CFU/mL). Error bars represent standard deviation (*n* ≥ 3). Statistical significance (*p* < 0.05) is illustrated by different letters a−d.

The effect of GSE on the antimicrobial properties of 2% (w·v^−1^) alginate crosslinked with 10% (*w*/*w* alginate) citric acid was assessed against *E. coli* (Figure 6a) and *S. aureus* (Figure 6b). The respective test items (83.3% *v*/*v*) were incubated with the cell suspension (≈10^7^ CFU/mL) at 37 °C and 300 rpm for 24 h. For control samples, a significant increase (*p* < 0.001) in the bacterial concentration and a decrease in pH (from 6.9 ± 0.2 and 7.0 ± 0.2 to 6.2 ± 0.2 and 6.4 ± 0.2, respectively, for *E. coli* and *S. aureus*) were observed after the incubation period.

Compared to the control samples, about 2.3- and 2.8-log_10_ reductions in viable counts of *E. coli* (*p* < 0.001) and *S. aureus* (*p* < 0.001), respectively, were observed in alg_ca10 samples after 24 h incubation at 37 °C. The observed inhibitory effect can be attributed to the effect of citric acid and the pH of the treatment solutions (4.2 ± 0.1), given the growth limits of *E. coli* (pH 4.3–10) and *S. aureus* (pH 4.5–9.3) [68] and the non-present antimicrobial activity reported for alginate [5,7]. Due to the differences in the cell wall structure, Gram-positive bacteria are more susceptible than Gram-negative to the inhibitory activity of citric acid, i.e., the disruption of substrate/electron transport through the permeabilised cell membrane due to cation chelation and the induction of intracellular reactive oxygen species leading to lipid peroxidation in the cell membrane [15,17,69,70,71].

The treatment solutions with aqueous GSE filtrate (GSE_alg_ca10) resulted in 2.8-log_10_ (*p* < 0.001) and 5.5-log_10_ (*p* < 0.001) reductions in viable counts of *E. coli* and *S. aureus*, respectively.

The TPC of the GSE used in this study was 0.22 ± 0.03 g gallic acid equivalent (GAE)/g GSE powder or 11.66 ± 1.32 g GAE/L GSE filtrate. According to the manufacturer, the GSE powder consists of 90% (*w*/*w*) polyphenols, including proanthocyanidins. The addition of GSE into a variety of films to improve antimicrobial properties has been broadly reported in the literature [26,27,29,30,72,73,74,75]. In particular, several authors [26,28,30] have reported a moderate (or even absent) inhibitory effect of GSE incorporated in different films on Gram-negative bacteria, as compared to Gram-positive, which was attributed to their lipidic cell wall acting as a barrier for polyphenols to enter the bacterial cytoplasm, and could also explain the differences observed in this work. Interestingly, Gadang et al. (2008) [73] reported the enhanced inhibition of *Listeria monocytogenes* in GSE-whey protein isolate films when adding 1% malic acid due to the decrease in the intracellular pH and enhanced susceptibility of the cell membrane to antimicrobial agents, which can be related to the synergistic effect of GSE, alginate, and citric acid found in this work, particularly for *S. aureus*.

#### 3.2.6. Effect of pH on the Colour of GSE-Based Film-Forming Solutions

In this section, the potential of GSE towards visually responsive (intelligent) food packaging materials was investigated, inspired by the patterned colour responses to pH variations reported in phenolic compounds [76,77].

**Figure 7:**
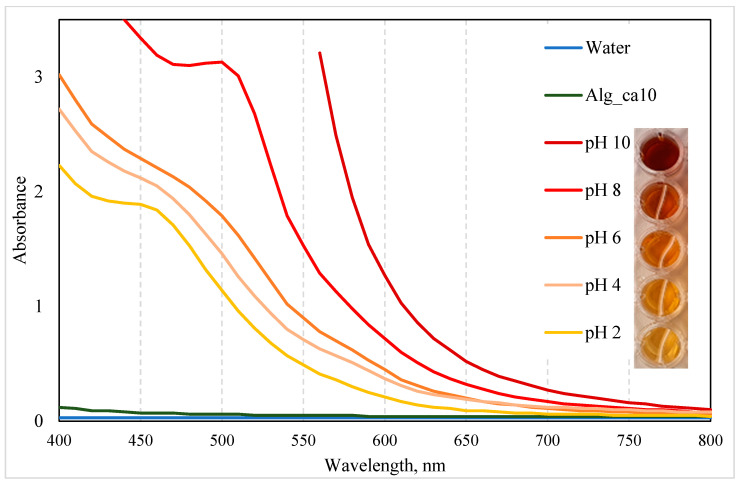
Effect of pH (2.0−10.0) on the UV/VIS spectra and colour of GSE-based film-forming solution (GSE_alg_ca10). Controls: water and alg_ca10 film-forming solutions at original pH values. The photograph on the right side presents the colour variations of GSE_alg_ca10 film-forming solutions at different pH levels.

The UV/VIS spectra of (undiluted) GSE_alg_ca10 film-forming solutions adjusted to pH 2.0–10.0 are presented in Figure 7. Absorption peaks were observed at 450 nm (GSE_alg_ca10 at pH 2.0), 490 nm (GSE_alg_ca10 at pH 8.0), and 500 nm (GSE filtrate at pH 10.0), although they were not clearly distinguishable for other tested pH values. Interestingly, the pH adjustment of the GSE_alg_ca10 film-forming solution from 2.0 to 10.0 shifted the UV/VIS spectra absorption peak from 450 to 500 nm, which resulted in a colour change from yellow to red. Guo et al. (2021) [31] attributed the shift in UV/VIS absorption peaks (278–502 nm) and the associated colour change (from orange-yellow to dark red) of proanthocyanidins, which, according to the manufacturer, are present in the GSE used in this study, to changes in their molecular structure as a result of the pH variation (3.0–11.0). In particular, due to the introduced protons or hydroxide radicals, the molecular polarisation increased, and the mobility of π electrons changed, influencing the variation in the colour of the compound.

## 4. Conclusions

In conclusion, the findings of this study have shown the potential of combining the natural active agent GSE with citric acid to enhance the functionality and bioactivity of alginate films for applications in smart food packaging. The incorporation of 10% citric acid as a crosslinking agent significantly increased the tensile strength of the alginate film and decreased the WVTR. With the further addition of GSE, the alginate films with 10% citric acid exhibited improved antioxidant and antimicrobial properties, while keeping good mechanical and barrier properties. The utilization of GSE in crosslinked films introduced a colour sensitivity to pH, which could be a promising feature for the development of intelligent packaging systems. These developed GSE-alginate-citric acid films have a potential to be used in the food industry to enhance food safety, quality, shelf-life, etc.

## Figures and Tables

**Table 1:** Overview of the composition and pH of film-forming solutions, as well as thickness and barrier properties of tested films. Statistical significance (*p* < 0.05) within a column is illustrated by different letters (a–e).

Sample Code	Composition (%w·v^−1^)				pH		Thickness (mm)	WVTR, (g/m^2^h)	
	Solvent	alg	SHP	CA					
alg	DW	2.00	-	-	5.9 ±	0.1 ^a^	0.04 ± 0.01 ^a^	138.56 ±	4.87 ^a^
alg_ca5	DW	2.00	0.05	0.10	4.4 ±	0.2 ^b^	0.04 ± 0.00 ^a^	98.49 ±	9.46 ^c^
alg_ca10	DW	2.00	0.10	0.20	4.0 ±	0.1 ^c^	0.04 ± 0.00 ^a^	91.99 ±	4.41 ^c^
alg_ca15	DW	2.00	0.15	0.30	3.8 ±	0.1 ^d^	0.05 ± 0.00 ^a^	89.66 ±	9.74 ^c^
alg_ca20	DW	2.00	0.20	0.40	3.7 ±	0.1 ^e^	0.05 ± 0.00 ^a^	78.04 ±	3.17 ^d^
GSE_alg	GSE	2.00	-	-	5.9 ±	0.2 ^a^	0.06 ± 0.01 ^b^	116.02 ±	5.16 ^b^
GSE_alg_ca10	GSE	2.00	0.10	0.20	4.2 ±	0.2 ^b^	0.07 ± 0.00 ^b^	91.49 ±	5.00 ^c^

alg: alginate, CA: citric acid, ca5: 5% (*w*/*w*) citric acid, ca10: 10% (*w*/*w*) citric acid, ca15: 15% (*w*/*w*) citric acid, ca20: 20% (*w*/*w*) citric acid, GSE: grape seed extract, SHP: sodium hypophosphite monohydrate, WVTR: water vapor transition rate.

## Data Availability

Not applicable.
